# Subcellular localization of Na^+^/K^+^-ATPase isoforms resolved by in situ hybridization chain reaction in the gill of chum salmon at freshwater and seawater

**DOI:** 10.1007/s10695-023-01212-6

**Published:** 2023-07-19

**Authors:** Marty Kwok Shing Wong, Yousuke Tsuneoka, Takehiro Tsukada

**Affiliations:** 1 Department of Biomolecular Science, Toho University, 2-2-1 Miyama, Funabashi, Chiba, 274-8510 Japan; 2 Center for Earth Surface System Dynamics, Atmosphere and Ocean Research Institute, the University of Tokyo, 5-1-5 Kashiwanoha, Kashiwa, Chiba, 277-8564 Japan; 3 Department of Anatomy, Faculty of Medicine, Toho University, 5-21-16 Omori-nishi, Ota, Tokyo, 143-8540 Japan

**Keywords:** Ionocyte, Salmonid, Osmoregulation, Morphometrics, Nonmetric multidimensional scaling (NMDS), In situ hybridization chain reaction (ISHCR)

## Abstract

The Na^+^/K^+^-ATPase (NKA) α1-isoforms were examined by in situ hybridization chain reaction (ISHCR) using short hairpin DNAs, and we showed triple staining of NKA α1a, α1b, and α1c transcripts in the gill of chum salmon acclimated to freshwater (FW) and seawater (SW). The NKA α1-isoforms have closely resembled nucleotide sequences, which could not be differentiated by conventional in situ hybridization. The ISHCR uses a split probe strategy to allow specific hybridization using regular oligo DNA, resulting in high specificity at low cost. The results showed that NKA α1c was expressed ubiquitously in gill tissue and no salinity effects were observed. FW lamellar ionocytes (type-I ionocytes) expressed cytoplasmic NKA α1a and nuclear NKA α1b transcripts. However, both transcripts of NKA α1a and α1b were present in the cytoplasm of immature type-I ionocytes. The developing type-I ionocytes increased the cytoplasmic volume and migrated to the distal region of the lamellae. SW filament ionocytes (type-II ionocytes) expressed cytoplasmic NKA α1b transcripts as the major isoform. Results from morphometric analysis and nonmetric multidimensional scaling indicated that a large portion of FW ionocytes was NKA α1b-rich, suggesting that isoform identity alone cannot mark the ionocyte types. Both immature or residual type-II ionocytes and type-I ionocytes were found on the FW and SW gills, suggesting that the chum salmon retains the potential to switch the ionocyte population to fit the ion-transporting demands, which contributes to their salinity tolerance and osmoregulatory plasticity.

**Supplementary Information:**

The online version contains supplementary material available at 10.1007/s10695-023-01212-6.

## Introduction

Due to the difference of osmotic gradients between the body fluid and environmental water, bony fishes gain water and lose ions in freshwater (FW) while they lose water and gain ions in seawater (SW) mainly through the body surface or gills (Lee et al. 2022). One of the main osmoregulatory strategies in fish is possessing ionocytes on the gill to transport ions, so that losing and gaining due to the osmotic challenges are countered (Hiroi and McCormick 2012; Cruz et al. 2013; Inokuchi et al. 2022). In ionocytes, Na^+^/K^+^-ATPase (NKA) is the main driving force for the ion-transporting and different isoforms of catalytic α-subunits were expressed in ionocytes of different salinities (Shrimpton et al. 2005; Nilsen et al. 2010; Bystriansky and Schulte 2011; Wong et al. 2016). Among different isoforms of NKA α-subunits, NKA α1a and α1b are famously reported as the Na^+^-pumps in the ionocytes of salmons acclimated to FW and SW, respectively (McCormick et al. 2009). Using specifically raised antibodies, NKA α1a and α1b proteins were differentially recognized in Atlantic salmon and successful Western blotting and immunohistochemistry were possible thereafter (McCormick et al. 2013, 2019). The gene expression of NKA α1a and α1b were measured by quantitative real time PCR (qPCR) and their expression levels were extensively examined in various fish species (Kaneko et al. 2002; Scott et al. 2004; Hiroi and McCormick 2012; Bollinger et al. 2016; Miyanishi et al. 2016; Inokuchi et al. 2022). In salmonids, a high expression of NKA α1a was generally associated to FW acclimation while NKA α1b expression was linked to a SW environment (Richards et al. 2003; Bystriansky et al. 2006; Tipsmark and Madsen 2009; Christensen et al. 2018; Wong et al. 2019). However, since the nucleotide sequences are highly similar between NKA α1a and α1b, conventional in situ hybridization (ISH) protocol that relies on labeled cRNA probes (200–1000 bp nucleotides) may not offer the specificity for distinguishing the two isoforms. So far, few studies have examined the transcript localization of NKA isoforms in fish except one study in Atlantic salmon has utilized small alkaline phosphatase (AP)-conjugated nucleotide oligos to achieve specific ISH of NKA α1a and α1b transcripts (Madsen et al. 2009).

Chum salmon is a Pacific salmon that migrates to the rivers of Northeast Japan for spawning in the winter. The salmon fries migrate to the sea after several months of FW life, and it was shown that the smoltification process occurs when the alevin emergence from the gravels (Wong et al. 2019). The chum salmon possesses high plasticity to salinity challenges and their plasma Na^+^ were relatively constant after direct transfer from FW to SW or vice versa. The branchial NKA activity in the chum salmon is higher than that of Atlantic salmon (McCormick et al. 1991), which may contribute to their excel capability in osmoregulation. Although the qPCR assays for the NKA α1a, α1b, and α1c have been developed in chum salmon, the localization of these isoforms in the ionocytes were unknown. In wild chum salmon, the expression of NKA α1a transcripts was high in FW environment while a lower expression was associated with SW environment (Nobata et al. 2022). On the other hand, NKA α1b expression was upregulated during smoltification and stayed at high levels among juveniles (Wong et al. 2019). The same study showed that the branchial expression of NKA α1c transcripts was not affected by salinity changes. The interplays among the NKA α1-isoforms in ionocytes are important to interpret the shifts in cell functions at different salinities (Richards et al. 2003). The knowledge of their cellular topology/localization and relative abundance may reveal differences in the osmoregulatory mechanism that allows rapid adjustment during salinity challenges in chum salmon.

To overcome the specificity barrier of closely resemble isoform in ISH, we tested a recently available technique known as in situ hybridization chain reaction (ISHCR), a method using short hairpin DNAs to localize the transcripts of NKA α1a, α1b, and α1c in the gill of chum salmon (Tsuneoka and Funato 2020; Katayama et al. 2022). This method allows multiple staining of closely resemble gene isoforms at the transcript level. The fluorescent signals of the target gene expression are quantitative, allowing downstream quantification analysis to measure the relative abundance of the NKA α1-transcripts in each ionocyte. To this end, we used a morphometric approach to understand the distribution of NKA α1-isoforms among the gill ionocytes of chum salmon acclimated to FW and SW.

## Materials and methods

### Animal husbandry

Fertilized eggs of chum salmon [*Oncorhynchus keta* (Walbaum, 1792)] were obtained from the Unosumai hatchery, Iwate, Japan. The artificially fertilized eggs were transferred to the Atmosphere and Ocean Research Institute, Chiba, Japan in Spring 2020. The embryos hatched in the recirculating FW system at 12°C. After emergence from gravel, juvenile salmon were fed with commercial diet specific for salmonids (protein: >46.0%, fat: >4.0%, fiber: >3.0%, carbohydrate: <15.0%, calcium: >1.5%, phosphate: >1.2% from the Marubeni Nishin Feed Company, Chita city, Aichi Prefecture, Japan). Some individuals were transferred to SW 4 weeks after emergence from gravel. They were reared for 2 years in an indoor aquarium facility at 16°C with a 14hL:10hD photoperiod until they reached 200–400 g. The water was aerated with air continuously to maintain dissolved oxygen and circulating filters were washed regularly to maintain water quality.

### Sampling

Five individuals (200–400 g) of each salinity were selected from FW or SW tanks. The individuals were not fasted before sampling. They were anaesthetized with 0.1% ethyl 3-aminobenzoate methanesulfonate (Sigma-Aldrich Chemicals, St Louis, MO, USA) neutralized with NaHCO_3_ (Carter et al. 2011). Fish were weighed, gill arches were excised and immediately fixed in 4% paraformaldehyde (PFA) in 0.1 M phosphate buffer at pH 7.4 for 5 h at 4°C. The fixed tissues were transferred to 70% ethanol until further processing.

### Tissue processing

Fixed gill arches were trimmed to remove gill arch while the filaments were dehydrated in graded ethanol, cleared in xylene, and embedded in paraffin (Pathoprep 568, Fujifilm Wako Pure Chemicals, Osaka, Japan) (Wong et al. 2016). No decalcification was performed. Paraffin sections at 20 μm and 4 μm were prepared by a microtome (Leica RM2125 RTS, Leica Biosystems, Tokyo, Japan) for ISHCR and histology. The sections were mounted on MAS-coated slides (Matsunami, Osaka, Japan) and stored at 4°C until use. MAS-coated slides are glass slides coated with proprietary materials by Matsunami company, which allows the tissue to attach to the glass slide firmly under harsh washing conditions including high temperature and extreme pH.

### Histology

The tissue sections (4 μm) were deparaffinized and rehydrated to deionized water by xylene and graded ethanol series. Standard hematoxylin and eosin staining protocol (H&E) was performed on the gill sections. Micrographs were taken by a CMOS camera (AdvanCam-U3; AdvanVision, Tokyo, Japan) equipped to a BX-53 microscope (Olympus, Tokyo, Japan) with an 100× oil-immersion objective lens.

### In situ hybridization chain reaction (ISHCR) using short hairpin DNAs

The ISHCR was modified from previous studies using short hairpin DNAs (Tsuneoka and Funato 2020; Katayama et al. 2022). Initiator sequences were assigned to NKA α1a (initiator 45), NKA α1b (initiator 41), and NKA α1c (initiator 73). The specific split probes with corresponding initiator sequences were designed on the mismatch regions from the nucleotide alignment (Supplementary Fig. 1). For each target, 5 sets of split probes were designed, and they were synthesized as standard oligos (Supplementary Table 1). The 5 sets of split probes were mixed and diluted to 10 μM, 2 μM, and 2 μM for NKA α1a, α1b, and α1c, respectively.

Paraffin sections (4 μm or 20 μm) were deparaffinized by xylene and hydrated to distilled water by graded ethanol series. They were treated with 3% H_2_O_2_ in methanol for 10 min at room temperature, washed by phosphate buffered saline with 0.1% Tween 20 at pH 7.6 (PBST) twice, and then equilibrated with a hybridization solution [5 × saline sodium citrate solution (SSC, Nacalai USA), 10% dextran sulfate (500,000; Fujifilm Wako Pure Chemicals), 30% formamide (Nacalai USA), 0.1% Tween 20 (Sigma-Aldrich, San Diego, CA, USA), and 11 U/mL heparin sodium (Nacalai USA)] in a moist chamber for 5 min at 37°C. The specific probe mix was denatured at 95°C for 5 min and then diluted to 100 nM (NKA α1a), 20 nM (NKA α1b), and 20 nM (NKA α1c) final concentrations in hybridization solution. The mixture was introduced to the sections and then spread evenly by covering with a sheet of parafilm. The hybridization was carried out at 37°C overnight in a moist chamber.

After the hybridization, the slides were washed 3 times with probe washing buffer (5 × SSC, 30% formamide, and 0.1% Tween 20, 10 min each) and 1 time with 5 × SSC for 10 min. After washing, the sections were bleached by an LED illuminator [TiYO, (Tsuneoka et al. 2022)] for 120 min in PBST to quench auto-fluorescence. Sections were then equilibrated with amplification buffer [8 × SSC, 10% dextran sulfate (MW 500,000), 0.2% Triton X-100 (Sigma-Aldrich), and 100 mM MgCl_2_] for 5 min. The fluorophore-conjugated hairpin DNAs provided by Nepagene (Chiba, Japan) were heated to 95°C for 1 min and gradually cooled to 65°C in the span of 15 min and to 25°C in the span of 40 min to allow the formation of hairpin structure. The hairpin DNAs were then diluted to 60 nM final concentration by amplification buffer with Hoechst 33342 (1 μg/ml; Dojindo Laboratories, Kumamoto, Japan) and applied onto the sections. Sections were incubated at 25°C for 2 h for the chain reaction development.

The chain reactions were stopped by washing the slides in PBST for 5 min at room temperature. Sections were mounted with antifade reagent (VECTASHIELD Vibrance, Vector Laboratories, H-1700). Negative control was performed with the same procedures except the probes were omitted in the hybridization step. Sections were observed by a confocal laser microscope (Nikon Eclipse Ti microscope equipped with the A1R confocal detection system) under 20×/0.75 or 60×/1.30 NA objective lenses. To obtain 3-dimensional image of ionocytes, *Z*-stack images were captured with a distance of 0.5 μm between the optical slices.

### Morphometric analysis

Since we found that neither the lamellar ionocytes nor the filament ionocytes expressed exclusively NKA α1a (green) or NKA α1b (red), we analyzed the ratio of the two isoforms with a morphometric approach. However, NKA α1c (magenta) was found to be ubiquitously expressed on the epithelial cells; thus, we excluded this isoform from the morphometric analysis.

Separate fluorescent channels were used to capture the images of NKA α1a (green) and NKA α1b (red) by an 20× objective to provide a broad area for analysis. Fluorescent intensities were adjusted to an undersaturated level for each channel for quantification purpose. Each slide was photographed under the same fluorescent setting to allow quantitative comparison. The fluorescent intensities were not adjusted by any image software before the quantification except when drawing the outlines of the ionocytes (see below). All fluorescent images presented in this study are untampered images.

Using ImageJ software, the fluorescent images of NKA α1a (green) and NKA α1b (red) were merged (Schneider et al. 2012). Intensity and contrast were adjusted to obtain a binary image that shows all ionocytes on the photo. Using the function of “Analyze Particles” with particle size from 100-infinity, the areas of interest (ROIs) of the ionocytes were assigned automatically. The cutoff value at 100 was optimized from trials and errors to prevents small particles or pillar cells from being grouped into the ROIs. Ionocytes that having area lower than 100 were excluded from the analysis. The areas of the ROIs were recorded, and then the ROIs were overlayed on the fluorescent images of NKA α1a (green) and NKA α1b (red), separately to quantify their intensities. The fluorescent intensity was normalized against the area of the ROIs to account for the size-dependent difference. The normalized data represent the differentiate NKA α1a and NKA α1b expressions in a single ionocyte.

### Statistics and meta-analysis

The ionocytes were grouped according to their area deduced from morphometric studies. In each salinity, the NKA α1a and NKA α1b fluorescent intensities per area were averaged according to the size categories (*N*=5), and two-way ANOVA followed by Tukey’s multiple comparison was performed to test for significant difference among the size-dependent expression of NKA α1 isoforms. Significant difference among groups was determined when *p*<0.05 was found.

Since FW ionocytes were found to express both NKA α1a and NKA α1b transcripts, we analyzed the distribution of NKA α1a and NKA α1b intensities, salinity, and ionocyte size, using nonmetric multidimensional scaling (NMDS). The NMDS analysis was performed with the R software v. 1.3.1093 (R core team). Permutational analysis of variance (PERMANOVA) in R software was performed to determine the effects of salinity on the distribution of NKA isoform intensities in the ionocytes (*p*<0.05). The salinity was set as a descriptive factor while cell area and relative abundance of isoform were set as variables. When the average intensity of NKA α1a transcripts was higher than that of NKA α1b, we defined the ionocyte as NKA α1a-rich ionocyte and vice versa.

## Results

**Fig. 1:**
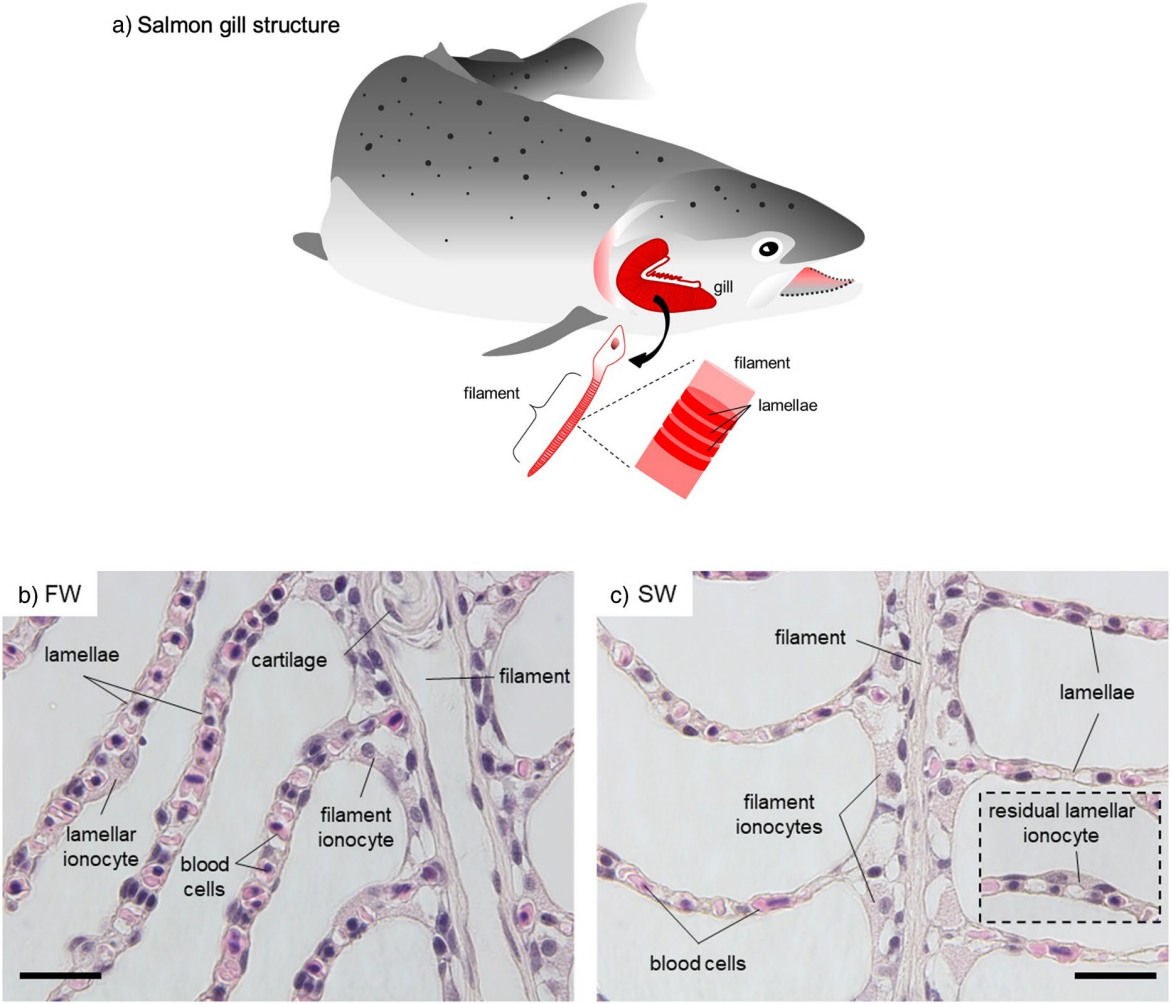
General structure and histology of chum salmon gill in FW and SW. Schematic diagram **a** shows the general structures of the gill in chum salmon. Each gill arch is composed of numerous filaments. Each filament has microscopic foldings known as lamellae that increase the surface area for gaseous exchange. Panels **b** and **c** show the hematoxylin and eosin (H&E) staining of gill sections from FW and SW, respectively. In FW (**b**), prominent lamellar ionocytes are present on the lamellae while small filament ionocytes are present at the interlamellar region. In SW (**c**), large filament ionocytes are found at interlamellar regions. Residual lamellar ionocytes are scarcely found on the lamellae. Micrographs were taken by an 100x objective. Scale bar = 10 μm

The morphologies between chum salmon gill in FW and SW are similar and each gill arch contains numerous filaments, which are further elaborated with secondary epithelium known as lamellae (Fig. 1a). The H&E staining shows the general histology of the gill epithelia in FW and SW chum salmon (Fig. 1b–c). Each filament is supported by a cartilage and lamellae are projected perpendicularly from the filaments. A single layer of epithelial cell is covering the lamellae, and a single line of blood cells can be observed in the capillary of each lamella. The majority of the ionocytes are found on the filament at the interlamellar regions. Large ionocytes could be found on the lamellae in elongated and flat shapes, blanketing some parts of the lamellar surface. In SW, the majority of ionocytes are found on the filament but few lamellar ionocytes were occasionally found (Fig. 1c). The filament ionocytes in SW are relatively larger than those in FW. The cytoplasm of ionocytes in both salinities appears to be granulated and not eosinophilic.

### In situ hybridization chain reaction of NKA α1-isoforms in gill ionocytes

**Fig. 2:**
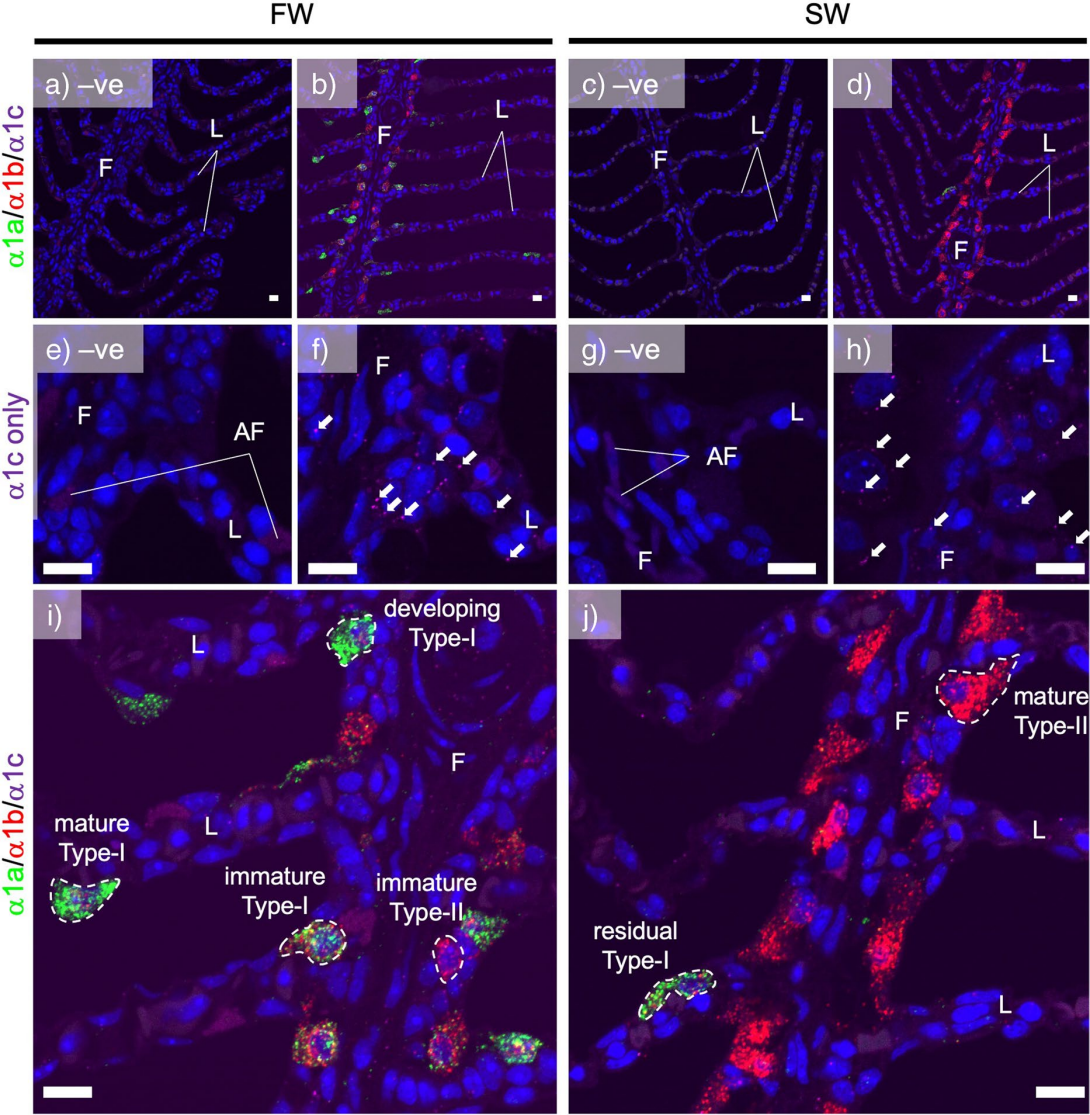
Differential localization of NKA α1-isoforms in the ionocytes in the gill of chum salmon acclimated in FW and SW. Panels **a** and **c** show the negative controls without probes, while panels **b** and **d** show the triple staining of α1a (green), α1b (red), and α1c (magenta) in the gills of acclimated individuals in FW and SW. The dot-type fluorescence was absent in negative controls, suggesting that non-specific signals are negligible. In FW (**b**), α1a and α1b positive ionocytes are found on the lamellae (L), while α1b-rich ionocytes are prominent on the filament (F). In SW (panel **d**), the major ionocytes are on the filaments and they are α1b-rich, with occasional observation of α1a-rich ionocytes. Panels **e** and **g** show the negative controls (no probes) while panels **f** and **h** show NKA α1c staining (magenta dots, indicated by white arrows) at high resolution in the gills of acclimated individuals in FW and SW, indicating that the NKA α1c is expressed in most cell types at low levels. Blood cell has residual auto-fluorescence (AF) that appears as even intensity signals. Magnified images of the gills of acclimated individuals in FW and SW (panels **i**–**j**) show the distribution characteristics of NKA α1-isoforms in type-I and type-II ionocytes at various stages. Specific staining appears as fluorescent dots where type-I ionocytes possess both green (α1a) and red (α1b) dots and type-II ionocytes possess mainly red dots (α1b). In FW (panel **i**), immature and developing type-I ionocytes were commonly found at the proximal region of the lamellae, and these cells possess both α1a and α1b transcripts. Immature type-I ionocytes are smaller and both α1a and α1b transcripts were found in the cytoplasm. In developing and mature type-I ionocytes, α1a transcripts were cytoplasmic while α1b transcripts were nuclear. Immature type-II ionocytes were small with prominent cytoplasmic α1b transcripts on the filaments in FW. In SW (panel **j**), type-II ionocytes with main cytoplasmic α1b transcripts were prominent on the filaments. Cytoplasmic α1a and nuclear α1b ionocytes were rarely found and they are considered as residual type-I ionocytes in SW. The objective of 20× and 60× were used for micrographs a–d and e–j, respectively. Scale bar = 10 μm

The ISHCR successfully distinguished the transcript localization of NKA α1a, α1b, and α1c in the gill of chum salmon. Negative control slides were without signal or with some auto-fluorescence (AF) in the blood cells (Fig. 2a, c, e, g). NKA α1c was found to be expressed in epithelial cells (Fig. 2b, d, f, h, arrows). To distinguish between AF from ISHCR signal, we should note that the hairpin DNA polymerization at the hybridized region results in a dot-type fluorescence (Fig. 2f, h), while AF has signals with even intensity, which happens mainly in blood cells (Fig. 2e, g). Although the signals of NKA α1c transcripts were relatively weak, signal comparison with negative control slides demonstrated its expression. In case of NKA α1c, no apparent signal intensity and localization differences were found between FW and SW; thus, it was not included in further analysis.

The transcripts of NKA α1a and α1b were differentially expressed in different types of ionocytes in chum salmon gills in FW and SW (Fig. 2i–j). In FW lamellae, the prominent ionocyte was large with flat- or dome-shapes (Fig. 2i, mature type-I ionocyte). They are solitary cells migrating on the surface of the lamellae. The NKA α1a transcripts were dominant in the cytoplasm while NKA α1b transcripts were accumulated in the nucleus. In some sections, these large ionocytes exhibited bean-shape, suggesting that the ionocyte was blanketing the curvature of lamellae (Fig. 2i). Many of these large cytoplasmic α1a/nuclear α1b ionocytes were found on the distal region of the lamellae. In the proximal region of the lamellae, developing type-I ionocytes were commonly found (Fig. 2i, developing type-I ionocytes), and these cells have less cytoplasm but exhibited the same cytoplasmic α1a/nuclear α1b pattern. At the apical region of the filament, we found small ionocytes that were rich in both NKA α1a and α1b transcripts, and both isoforms were found within the cytoplasm and nucleus (Fig. 2i, immature type-I ionocytes). We expected them to be the immature type-I ionocytes for their isoform characteristics that are similar to the mature type-I ionocytes. The vicinity of these ionocytes suggested that the large lamellar type-I ionocyte was developed from the immature type-I ionocyte on the filament by cytoplasmic enlargement and redistribution of NKA α1a and NKA α1b transcripts. No progenitor or immature type-I ionocytes were found on the distal region of the lamellae.

Besides the immature type-I ionocytes, another type of ionocyte was observed on the filament in FW gill. At the basal region of the filament, small ionocytes that are rich in cytoplasmic NKA α1b were abundant (Fig. 2i, immature type-II ionocytes). However, NKA α1a signal was not found in the nucleus and was relatively low compared to that of NKA α1b in the cytoplasm. These cells were small with relatively thin cytoplasm, and they were mostly found at the basal region of the filament. We designated these cells as immature type-II ionocytes for their transcript characteristics that are similar to those of mature type-II ionocytes in SW gill (see below).

In SW gill, the major ionocytes were found to be embedded on filament and they were large with prominent NKA α1b expression (Fig. 2j, mature type-II ionocytes). NKA α1a transcript was found at low level but it is only observable in unmerged photos. Besides major type-II ionocytes, few type-I ionocytes were observed on the lamellae and on the apical region of filament (Fig. 2i, residual type-I ionocyte). However, these cells are scarce, and thus we expected that they are residual lamellar type-I ionocytes.

**Fig. 3:**
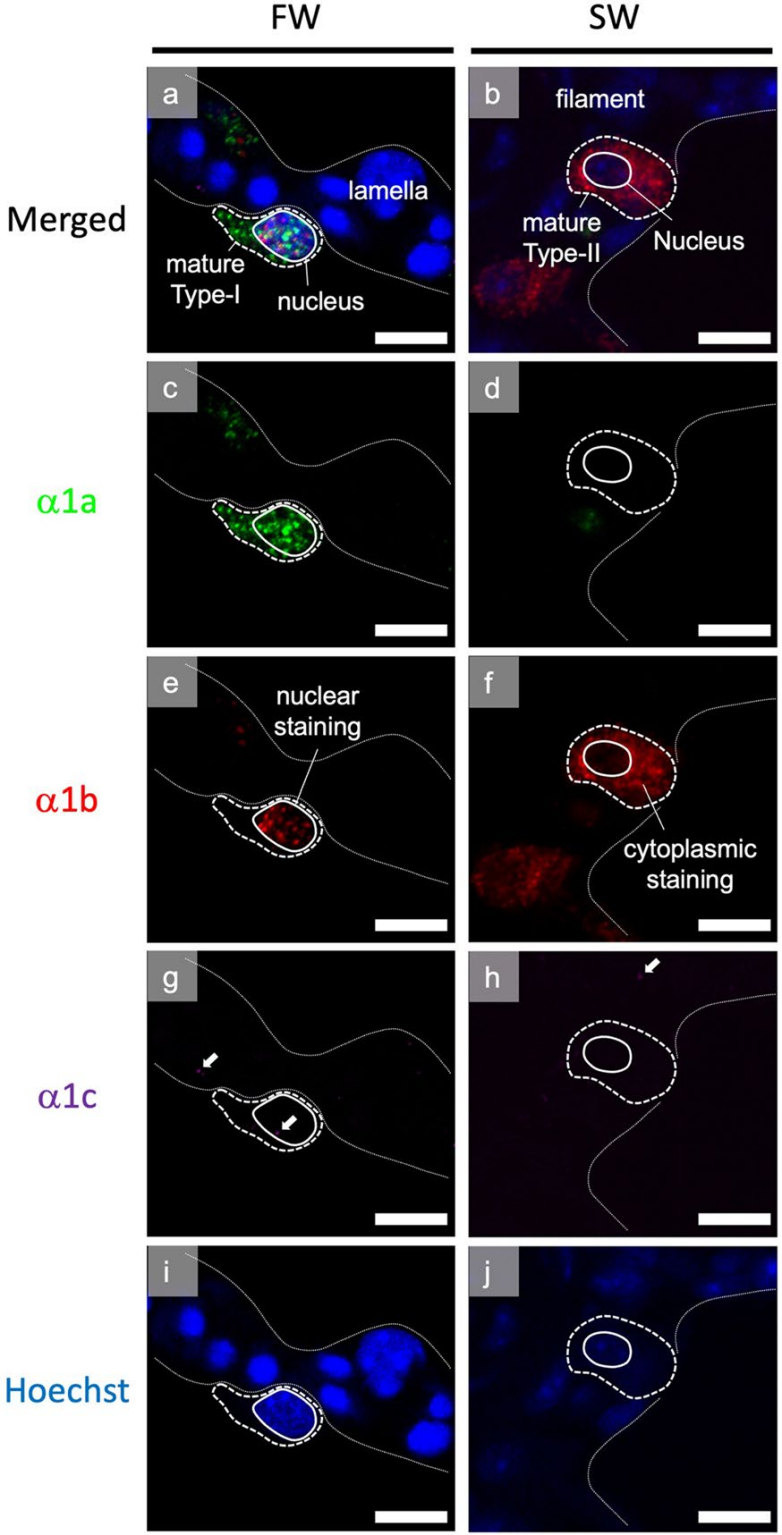
Characteristics of subcellular localization of NKA α- isoforms in type-I in FW and type-II ionocytes in SW. Panels **a** and **b** show the merged images of mature type-I and type-II ionocytes on the FW lamella and SW filament, respectively. Separate panels show the different fluorescent channels corresponding to α1a (**c**–**d**, green), α1b (**e**–**f**, red), α1c (**g**–**h**, magenta), and Hoechst (**i**–**j**, blue). Mature type-I ionocyte is characterized by cytoplasmic α1a and nuclear α1b transcript staining, while mature type-II ionocyte is characterized by cytoplasmic 1b without nuclear α1 transcript staining. NKA α1c transcripts are found as magenta dots, indicated by white arrows. Micrographs were taken by an 60× objective. Scale bar = 10 μm

The triple staining allows the subcellular localization of the 3 NKA α1-isoform transcripts in ionocytes. With the *Z*-stacking of confocal imaging, the 3-dimensional images of a lamellar type-I ionocyte in FW and a filament type-II ionocyte in SW were reconstructed (Supplementary files 1-2). From the separated fluorescent channels, we found that the transcripts of NKA α1a were abundant in both cytoplasm and nucleus in lamellar type-I ionocyte in FW, while NKA α1b was mostly accumulated in the nuclear region (Fig. 3c, e). In SW, filament type-II ionocyte expressed abundant NKA α1b and transcript were mainly found in the cytoplasm (Fig. 3d, f). The transcripts of NKA α1c were not accumulated in the ionocytes in both FW and SW, and scattered NKA α1c transcripts could be found in most cell types (Fig. 3g–h). The cytoplasmic α1a/nuclear α1b characteristic can be used as a marker for differentiating the type-I and type-II ionocytes in the gills of chum salmon in different salinities.

### Morphometrics and meta-analysis

**Fig. 4:**
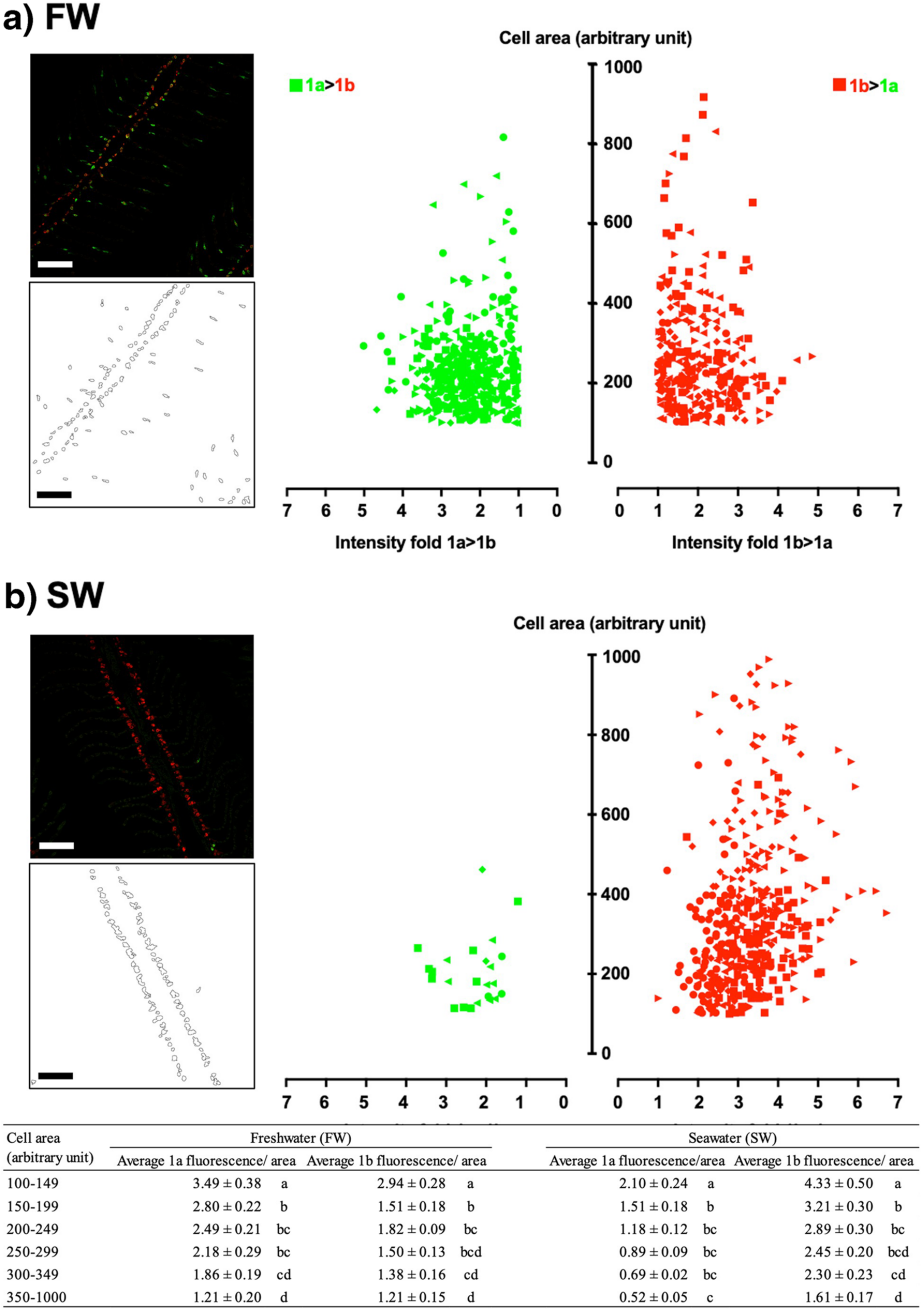
Volcano plots showing the size-dependent expression of NKA α1a and α1b in the gill of chum salmon acclimated to FW (panel **a**) and SW (panel **b**). Cells with average NKA α1a intensity higher than NKA α1b intensity are designated as green, and the vice versa are designated as red. Different symbol shapes data obtained from different individuals (*N* = 5 in each salinity). The inserted panels on the left show typical examples of morphometric analysis in which the regions of interest (ROIs) in the outlined diagrams are overlayed on the fluorescent image for quantification of area and intensity. Micrographs were taken by an 20× objective. Scale bar = 100 μm. Embedded table at the bottom shows the summary of meta-analysis of morphometric data. Average NKA α1a or α1b fluorescent intensity per ionocyte area was compared according to cell size. Data are in mean ± SEM (*N* = 5). The ionocytes were grouped according to different sizes and their fluorescent intensities were compared among size-dependent groups by two-way ANOVA, Tukey’s comparison (*p*<0.05, indicated by different letters)

The NKA α1a and α1b transcript abundance were analyzed quantitatively with morphometric methods. ROIs of ionocyte was generated by ImageJ program and cell area was measured (see examples in Fig. 4). The fluorescent intensity of NKA α1a and α1b transcripts were quantified and expressed as intensity per unit cell area. When the intensity of NKA α1a transcripts was higher than that of NKA α1b transcripts in the same ionocyte, it was considered as an NKA α1a-rich ionocyte and vice versa. The relationship between cell area and the transcript ratio of NKA α1a/α1b in NKA α1a-rich cells or the transcript ratio of NKA α1b/α1a in NKA α1b-rich cells were presented in volcano plots. In FW, the abundance of NKA α1a-rich cells and NKA α1b-rich cells were similar, and no obvious size-dependency was observed (Fig. 4a). The abundance of NKA α1b-rich cells was higher than that of NKA α1a-rich cells in SW. In addition, the size of NKA α1a-rich cells was relatively small in SW (Fig. 4b). Significant decreasing trends of fluorescent intensity per cell area were observed in both FW and SW ionocytes (Fig. 4, embedded table). NKA α1a expression was at relatively low levels in the NKA α1b-rich cells in SW, indicating that NKA α1b expression was dominant, but not exclusive, in type-II ionocytes.

**Fig. 5:**
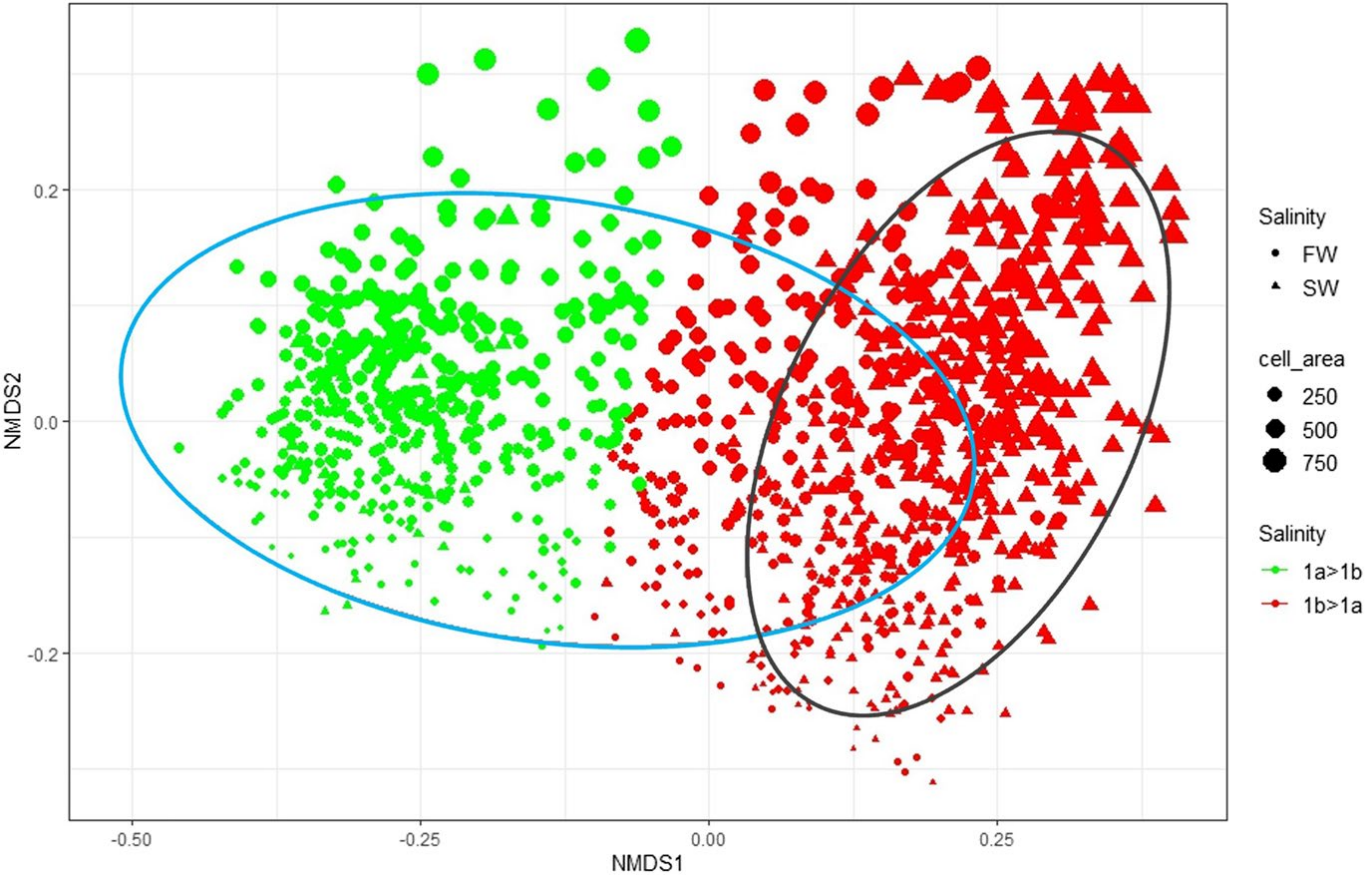
Nonmetric multidimensional scaling (NMDS) analysis of salinity, ionocyte size, and fluorescent intensity of NKA α1a and α1b in the gill of chum salmon acclimated to freshwater (FW) or seawater (SW). The ovals indicate the integrated centroids of FW (blue) and SW (dark gray) data. A large proportion of FW ionocytes are including NKA α1a-rich and NKA α1b-rich as shown by the blue oval while most SW ionocytes expressed relatively high level of NKA α1b

To combine the observations among cell size, salinity, and fluorescent intensities of NKA transcript isoforms, we used a multivariate regression method to analyze the distribution pattern of the data (Fig. 5). The NMDS aims to map the original position of the data in multidimensional space using a reduced number of dimensions. The NMDS result indicated that the NKA α1a-rich cells and the NKA α1b-rich cells were not separated by salinity effect as the distribution appears to be continuous between the two groups. The PERMANOVA result suggested that salinity effect was significant (*p*<0.001) on the distribution as ionocytes as they form different groups between FW and SW (Fig 5, blue oval—FW; black oval—SW). FW ionocytes consisted of both NKA α1a-rich and NKA α1b-rich cells, regardless of cell size. Among the 736 FW ionocytes in the morphometric analysis, 39.5% were NKA α1b-rich cells. On the other hand, among the 449 SW ionocytes in the morphometric analysis, 94.0% were NKA α1b-rich cells.

## Discussion

The description of NKA α1a and α1b in Atlantic salmon in FW and SW has grounded a foundation of salinity-specific isoforms in fish osmoregulation studies (McCormick et al. 2009). Many follow-up studies on salmonid osmoregulation have supported a model that NKA α1a is for FW while NKA α1b is for SW (Kiilerich et al. 2007; Tipsmark and Madsen 2009; Hiroi and McCormick 2012). The model was extrapolated to other species including tilapia and medaka, but the same model was not always applicable in teleosts since different lineages could have selected different NKA α1-isoforms as major forms in gill and other osmoregulatory epithelia during evolution (Wong et al. 2016). Within salmonids, chum and pink salmon are some diadromous migrating species with short FW juvenile lives (Stefansson et al. 2008). The salinity tolerance of chum salmon alevins and juveniles is high, and we expected that their ionocytes may possess special characteristics to cope with sudden salinity changes (McCormick 2012; Wong et al. 2019). To establish a basic framework of chum salmon ionocytes, we examined the transcript localization of the NKA α1-isoforms in the ionocytes in FW and SW environment. Our data suggests that NKA α1b expression could be partially redundant in FW ionocytes of chum salmon.

### Simultaneous staining of NKA α1-isoforms

Three NKA α1-isoforms were reported in chum salmon previously (Wong et al. 2019), and we examined their localization simultaneously with triple staining using ISHCR. NKA α1c was found to be expressed ubiquitously in most cell types and no salinity effect was observed (Fig. 2e–h), which matched with the qPCR data reported in the previous study (Wong et al. 2019). NKA α1a and α1b were found in the ionocytes in both FW and SW environments.

**Fig. 6:**
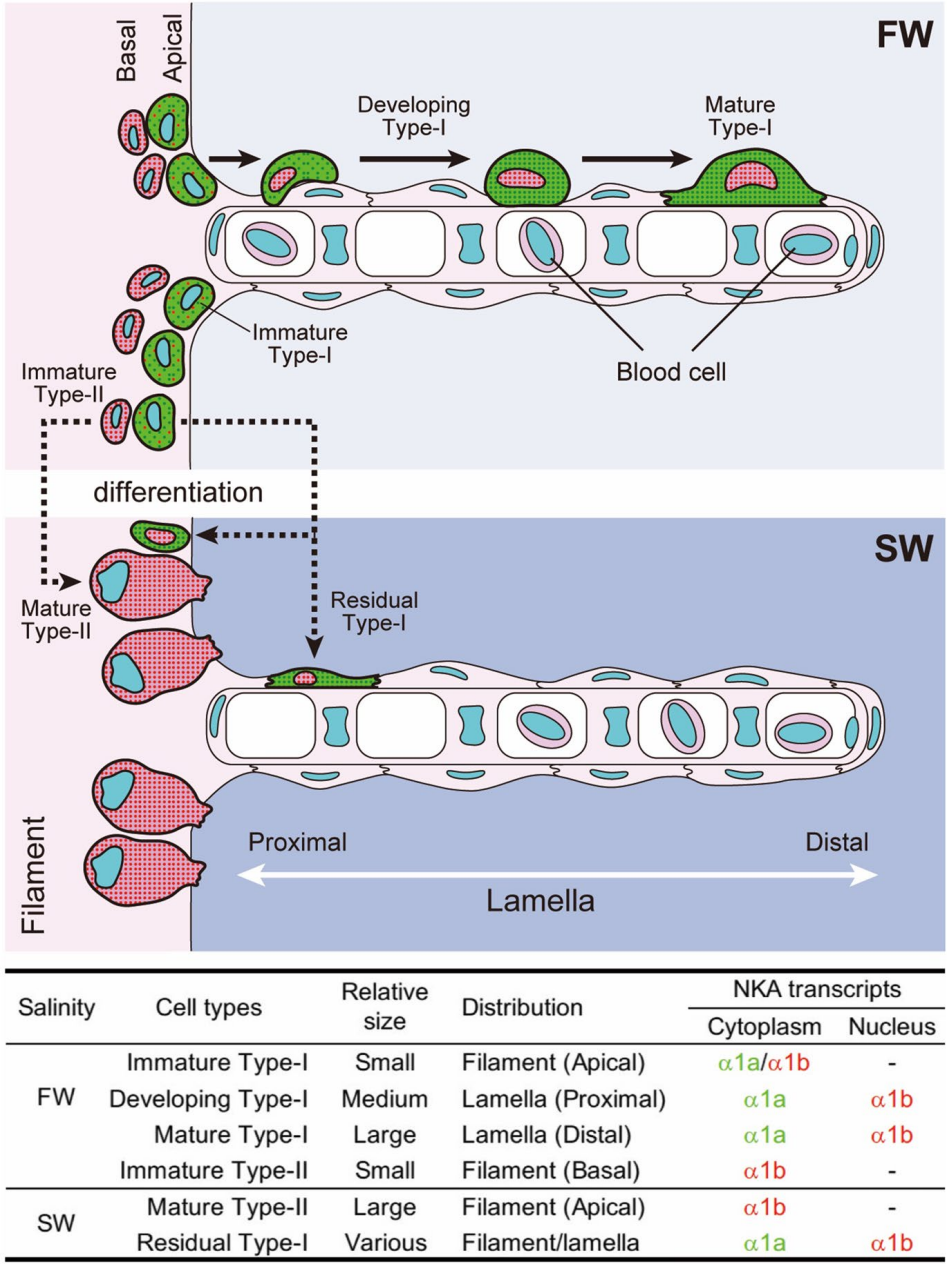
A schematic diagram summarizing the expression of NKA α1a and α1b expression in different types of ionocytes in chum salmon acclimated to FW and SW. In FW gill, two types of ionocytes are found on the filaments. One type is characterized by showing the expression of both NKA α1a and α1b while the other type is characterized by showing main NKA α1b expression. The former is developed into mature type-I ionocytes with cell enlargement, cytoplasmic accumulation of NKA α1a transcripts, and nuclear accumulation of NKA α1b transcripts. The mature type-I ionocytes migrate to the distal regions of the lamellae. In SW, the type-II ionocytes are supposed to be developed from the basal NKA α1b-rich cells and they are localized in the interlamellar region on the filament. Some type-I ionocytes are rarely found on the filaments or lamellae and they are thought to be some residual production. Embedded table compares the characteristics of different ionocytes types between the gill of FW and SW acclimated chum salmon. The relative cell size, localization, and NKA α1 transcript characteristics of type-I and type-II ionocytes are summarized. Different color of α1a and α1b are matching the fluorescent signals in this study

In the gill of FW acclimated individuals, we expected that the development of type-I ionocytes follows a sequence of NKA isoform redistribution. The vicinity of mature type-I ionocytes on the lamellae and immature type-II ionocytes on the filament suggested that the former migrate to the distal region of the lamellae after development (Fig. 6). No ionocyte progenitors were found on the lamellae of chum salmon (Uchida and Kaneko 1996), and morphological and histological examinations suggested that lamellar ionocytes are migrated from the filaments in seabass and air-breathing fishes acclimated to FW or hypo-osmotic conditions (Hirai et al. 1999; Lin and Sung 2003). Ionocyte migration was also observed in the interlamellar cell mass after hypoxia treatment in goldfish (Tzaneva et al. 2014). In addition, our observation did not identify any progenitor, immature, or developing NKA α1a-rich ionocytes on the distal end of the lamellae. Although the migration of type-I ionocytes was not directly demonstrated, the lack of evidence that mature lamellar ionocytes could be originated from the lamellae supports the migration scenario.

The immature type-II ionocytes on the filament contain rather less cytoplasm and both NKA α1a and α1b transcripts could be found in the cytoplasm, suggesting both isoforms were normally translated into proteins. During the ionocyte maturation, the cytoplasmic volume increased dramatically, and NKA α1b transcripts were confined in nucleus while NKA α1a transcripts were distributed in the cytoplasm. The nuclear localization of NKA α1b transcripts suggested that the gene was transcribed but it may not be properly translated into protein, thus leaving unprocessed transcripts at the nucleus (Hildyard et al. 2020). The functional shift from both NKA isoforms into NKA α1a as the major sodium–potassium ATPase pump implies different mechanism of ion-transporting between lamellar and filament type-I ionocytes. Although the detail changes in the ion transportation are not deduced, the cytoplasmic-α1a/nuclear-α1b feature is a useful morphological marker for distinguishing different types of ionocytes. In addition, another type of ionocytes that expressed mainly NKA α1b were found at the basal region of the filament. This type of ionocyte has an expression pattern that resembles that of SW, and we suggested that these are immature type-II ionocytes, which will be differentiated into functional type-II ionocytes with cytoplasmic enlargement when the fish encounters a SW condition (Fig. 6).

In SW, the major ionocytes are localized on the filaments, which have a large size with rich cytoplasmic NKA α1b. These ionocytes are typical in SW and they are known as chloride cells historically (Zadunaisky 1996). Few cytoplasmic-α1a/nuclear-α1b cells were found on the lamellae and filaments, and these could be the residual type-I ionocytes that have not been completely switched off from production. The important observation is that either in FW or SW, immature/residual type-I and type-II ionocytes exist, suggesting that production of different ionocytes never ceased in the gill of chum salmon, which guarantee the gill has the potential to reorganize the ion-transporting epithelium and/or mechanism when salinity challenges occur. We emphasize that the chum salmon used in the present experiments did not experience frequent fluctuation in salinities (e.g., tidal zone or estuary). The FW individuals have their whole lives in FW while the SW individuals were kept in full strength SW for over 2 years. Therefore, the capacities and redundancy of immature/residual type-I and type-II ionocytes in FW or SW show that the chum salmon may always be prepared to salinity challenge, even long after smoltification that occurs at alevin stage. The presence of reserve ionocytes was shown previously in medaka following repeated FW to SW transfers (Miyanishi et al. 2016).

An interesting phenomenon is that the lamellar type-I ionocytes in FW express cytoplasmic-α1a/nuclear-α1b transcripts. From the colocalization result (Fig. 2i), we expected that the immature or filament type-I ionocytes could be producing both NKA α1a and α1b proteins, suggesting that both forms may have roles in ion-absorption. However, the NKA α1b transcripts were found in the nucleus among mature type-I ionocytes, suggesting that active translation of NKA α1b may not be significant. Therefore, we expect that the major NKA α1 protein in the mature type-I ionocytes should be NKA α1a, which agrees with the immunolocalization results in other salmonids (Richards et al. 2003; Shrimpton et al. 2005; McCormick et al. 2009, 2019; McCormick 2012; Christensen et al. 2018). One possibility is that the NKA α1a and α1b transcription could have shared the same set of transcription factors; thus, the transcription regulation was common (Li and Langhans 2015; Wang and Chen 2019). For predominant production of NKA α1a protein in the type-I ionocytes, the production of NKA α1b protein could be suppressed by mRNA processing or translation inhibition. However, the hypothesis requires the proof from a promoter analysis of NKA α1a and α1b genes in chum salmon. Under the hypothesis, the resultant major NKA α-subunit in FW is NKA α1a protein even though the expression of NKA α1b is high (expressed but not translated). This also explain why the qPCR result showed high NKA α1b expression in both FW and SW, while the expression of NKA α1a was more sensitive to upregulation and downregulation by salinity changes in chum salmon (Wong et al. 2019; Nobata et al. 2022). On the other hand, we hypothesized that the transcription of NKA α1b could be regulated independently from that of NKA α1a; thus, only NKA α1b transcripts were found in type-II ionocytes. Future research on the promoter characteristics on the of NKA α1a and α1b genes will bridge up the gap of our understanding between transcription and translation of the two genes. However, we emphasized that the type-II ionocytes were not expressing NKA α1b exclusively, as the results from separate fluorescent quantification showed considerable expression of NKA α1a in type-II ionocytes (Fig. 4, embedded table), which was mostly masked by the overwhelm expression of NKA α1b. So far, there was only one ISH study on salmonid NKA α1-isoforms (Madsen et al. 2009), but we demonstrated triple staining of the NKA α1-isoforms for the first time. In the previous study using alkaline phosphatase (AP)-conjugated oligos (Madsen et al. 2009), the signal intensity would be difficult to control at sub-saturated levels for quantification purpose. Moreover, colocalization of α1a and α1b was not performed in previous study but we showed the subcellular localization of α1a and α1b transcripts, which led to the observation of the differential localization of α1a and α1b transcripts in the cytoplasm and nucleus in type-I ionocytes. Although we have not demonstrated the protein localization of NKA α1-isoforms in the gills of chum salmon, our result is a link to bridge the understanding between overall isoform expression from qPCR results and the distribution of the isoform transcripts at tissue and cellular levels. Further studies on the transcript distribution of NKA α1-isoforms in other salmonids using ISHCR may reveal whether the localization and isoform distribution was common in other species.

### Meta-analysis on morphometrics

Besides using a qualitative approach to localize the transcripts of NKA α1a and α1b, a semi-quantitative approach to measure their expression in a single ionocyte was performed. We also used a morphometric approach to examine the global changes in several individuals to ensure the results were not biased. The morphometric results were combined in a multivariate analysis to obtain a statistically supported pattern that inspires future studies. With NMDS analysis, we demonstrated that the ionocytes in FW and SW could not be divided solely by the dominant NKA α1-isoform as NKA α1b-rich ionocytes were detected in a large portion in FW acclimated fish gills (Fig. 5). We also realized that the cell locations are not definite for their putative functions as most of the FW ionocytes are on the filaments. Since we do not have the colocalization results of other transporters such as sodium-hydrogen exchangers (NHEs), sodium–potassium–chloride cotransporter 1 (NKCC1), sodium-chloride cotransporter (NCC), cystic fibrosis transmembrane conductance regulator (CFTR), to determine the exact roles of the ionocytes, the ionocyte names were designated as type-I and type-II ionocytes only at this stage.

### Ionocyte subtypes

In rainbow trout, previous research distinguished different types of ionocytes based on the binding of peanut lectin agglutinin (PNA) in intact gill and isolated ionocytes (Goss et al. 2001; Galvez et al. 2002; Reid et al. 2003). Using isolated cells, it was reported that PNA+ cells contributed to 35% and 80% of the ionocytes in FW and SW, respectively (Hawkings et al. 2004). While the PNA+ ionocytes were major in SW, the NKA activities in these cells decreased by 57%, suggesting that the PNA+ ionocytes may be different from the NKA-rich ionocytes typically found on the gill filaments since ionocytes in SW were consistently possess higher NKA activities in teleosts (McCormick 2012; McCormick et al. 2019). The percentage of PNA+ cells in FW rainbow trout varied due to various factors including tissue/cell preparation, types of ionocytes markers, and different trout populations described previously (Brannen and Gilmour 2018). However, most PNA cells were shown to possess high cytosolic carbonic anhydrase and their numbers increased under low pH, hypoxia, and hypercapnia conditions in rainbow trout (Gilmour and Perry 2009; Brannen and Gilmour 2018), suggesting that they are responsible for acid-base regulation. Despite the prominent use of PNA in differentiating different ionocytes, the α1-subunit types were not studied simultaneously. Further studies using different cell markers (e.g., PNA or other lectins) in conjunction to NKA α1-subunit markers (immunohistochemistry or ISHCR), and colocalization of different types of apical transporters (e.g., NHE3a/3b, CFTR) will enrich our understanding on the ionocyte dynamics, and further differentiate the cell types and functions accurately.

### Perspectives on ISHCR in fish studies

Teleosts experienced the 3R whole-genome duplication, resulting in many closely resemble genes that impose technical challenges to the researchers (Volff 2005). While the gene expression could be examined by specific qPCR that relies on a few nucleotide polymorphisms between gene isoforms, localization by conventional ISH using cRNA probes may not have sufficient specificities. To achieve specific hybridization of closely resemble genes, the split probes should be designed on the mismatch regions of the alignment (see Supplementary Fig. 1 for example). The first split probe is complementary to the first half of the target region while possessing a 3′-overhang. The second split probe possesses a 5′-overhang followed by the complementary sequence to the second half of the target region. When the two split probes hybridize on the target region, the two overhangs combine to form a complete initiator sequence that will activate a chain reaction when the initiator encounters the fluorophore-containing hairpin pairs. The hairpin pairs are designed in a way that the hairpin-1 can bind on the initiator sequence to open the hairpin region, exposing the hidden initiator sequence for hairpin-2. When the hairpin-2 binds on the hidden initiator sequence, the initiator sequence for hairpin-1 is then exposed. The alternative openings of hairpin-1 and hairpin-2 result in a chain reaction of polymerization of the hairpin pairs, which leads to strong fluorescent signals being accumulated at the hybridized region. By manipulating different initiator sequences and hairpins containing different fluorophores, colocalization of target genes can be achieved.

The short hairpin ISHCR protocol does not require both heating and proteinase K treatment, since the short DNA probes and hairpins can efficiently penetrate the tissue that was heavily crosslinked by paraformaldehyde fixation (Tsuneoka and Funato 2020). Genes with few nucleotide polymorphisms could be effectively distinguished since the split probe method used in the ISHCR provides a higher specificity when it requires the binding of two oligos adjacent to each other for starting the chain reaction (Choi et al. 2018). The initiator sequences for hairpin DNA used in this study were designed to have no off-target bindings in mouse genomes (Tsuneoka and Funato 2020). However, as most fish models may not have comprehensive genome information, negative controls with only hairpin DNA are important to test for possible off-target bindings. With the quenching technique by high-power LEDs to reduce auto-fluorescence, genes expressed at low copy number could be also detected (Tsuneoka et al. 2022). The ISHCR method is a revolutionary asset for fish physiological research as it provides high specificity at a lower cost compared to traditional ISH based on labeled cRNA probes.

## Supplementary information

Supplementary file 1 Supplementary Fig. 1. Nucleotide alignments of NKA α1a, α1b, and α1c cDNA showing regions where split probes were designed (boxed). The nucleotide used for split probes were underlined and the mismatched nucleotides were highlighted. (PDF 67 kb)

Supplementary file 2 Supplementary File 1. Z-stacked composite image of a Type-I ionocyte in the gill of freshwater (FW) chum salmon. When the file is opened with ImageJ program, the 3-dimensional image of the NKA α1a (green), α1b (red), and α1c (magenta) of the Type-I ionocyte can be observed. Nucleus is stained with Hoechst (blue). (TIF 28842 kb)

Supplementary file 3 Supplementary File 2. Z-stacked composite image of a Type-II ionocyte in the gill of seawater (SW) chum salmon. When the file is opened with ImageJ program, the 3-dimensional image of the NKA α1a (green), α1b (red), and α1c (magenta) of the Type-II ionocyte can be observed. Nucleus is stained with Hoechst (blue). (TIF 28518 kb)

Supplementary file 4 Supplementary Table 1. Split probes for NKA α1-isoforms in chum salmon. Initiator sequences for hairpin are underlined. (DOCX 17 kb)
