# Supplementary material for: Subcellular localization of Na+/K+-ATPase isoforms resolved by in situ hybridization chain reaction in the gill of chum salmon at freshwater and seawater
Source: Fish Physiol Biochem. 2023 Jul 19;49(4):751–67. doi: 10.1007/s10695-023-01212-6 (PMC10415477; doi:10.1007/s10695-023-01212-6)
Supplement: Supplementary file 1 — Supplementary Fig. 1. Nucleotide alignments of NKA α1a, α1b, and α1c cDNA showing regions where split probes were designed (boxed). The nucleotide used for split probes were underlined and the mismatched nucleotides were highlighted. (PDF 67 kb) [file 10695_2023_1212_MOESM1_ESM.pdf]

```
1a      TGTGGTTCGACAACCAGATCCATGACGCTGACACCACAGAGAACCAGAGTGGTACCTCTT
1b      TGTGGTTCGACAACCAGATCCATGAGGCTGACACCACAGAGAACCAGAGTGGTACCTGCT
1c      TGTGGTTCGACAACCAGATCCATGAGGCTGACACCACAGAGAACCAGAGCGGTACCTCCT
        ************************* *********************** *******  *

1a      TCGACAAAAGCTCTGCAACCTGGGCTGCCCTGGCTAGAGTCGCCGGCCTGTGCAACCGAG
1b      TCGACAAAAGCTCTGCCACCTGGGCTTCCCTGGCAAGAGTCGCTGGCCTGTGCAACCGCG
1c      TCGACAGGAGCTCTGCCACCTGGGCTGCCCTGGCTAGAGTCGCTGGCCTGTGTAACCGTG
        ******  ******** ********* ******* ******** ******** ***** *

1a      CCGTCTTCCTGGCAGAACAGAACAACGTACCTATCCTCAAGAGAGATGTGAGCGGTGATG
1b      CCGTCTTCCTGGCAGAACAGAACAACGTACCTATCCTCAAGAGAGATGTGGCTGGTGATG
1c      CAGTCTTCCTGGCAGAACAGAGCGGTATTCCTATCCTTAAAAGAGATGTGGCTGGTGATG
        * ******************* *    * ******** ** *********   *******

1a      CCTCAGAGACTGCCCTGCTGAAGTGTATCGAGCTGTGCTGTGGGTCTGTCAAAGACATGA
1b      CTTCAGAGTCTGCCCTACTGAAGTGTATCGAGTTGTGCTGCGGGTCTGTCAAAGACATGA
1c      CCTCAGAGTCTGCCCTGCTGAAGTGTATTGAGCTGTGCTGTGGGTCTGTGCAAGGCATGA
        * ****** ******* *********** *** ******* ********  *** *****

1a      GAGAAAAGTACAGCAAAGTCGTTGAGATCCCCTTCAACTCCACCAACAAATACCAGCTCT
1b      GAGAAAAGTACAGCAAGATCGCTGAGATTCCCTTCAACTCCACCAACAAATATCAGCTCT
1c      GAGACCAGTACACCAAGGTTGCTGAAATCCCATTCAACTCCACCAACAAATACCAGCTCT
        ****  ****** ***  * * *** ** ** ******************** *******

1a      CCATTCATGAGGACAGCACGGCCGGCGAGTCCAATCATCTGCTGGTGATGAAGGGAGCCC
1b      CCATTCATAAGAACATCGTGGCCGGAGAGTCCAATCACCTGCTGGTGATGAAGGGAGCCC
1c      CCGTTCACCTGAACAAGAATGAGGGTGAGTCCAAGCATCTGCTGGTGATGAAGGGAGCCC
        ** ****   * ***     *  ** ******** ** **********************

1a      CTGAGAGGATCCTGGACAGCTGCTCCACCATCTTGCTCCAAGGCAAAGAACATCCTCTGG
1b      CTGAGAGGATTCTGGATCGCTGCTCCACCATTTTGATCCAGGGCAAAGAACAGACTCTCA
1c      CTGAGAGGATTCTGGACCGCTGCTCCACCATTTTGATCCAGGGCAAAGAACAGCCTCTGG
        ********** *****  ************* *** **** ***********  ****

1a      ATGA CGAGATAAAGGAATCATTTCAGAAAGCCTACGAAGCGCTAGGAGGACTGGGA GAGA
1b      ATGA CGAGCTGAAGGAAGCCTTCCAGAACGCTTATGAAGAGCTGGGAGGGCTGGGA GAGA
1c      ATGA CGAGATGAAGGACTCTTTCCAGAACGCCTACATGGAACTGGGTGGTCTGGGA GAGC
        **** **** * *****  * ** ***** ** **    *  ** ** ** ****** ***

1a      GAGTGCTGGGTTTCTGCCATTTCCAGCTCCCTGATGACCAGTTTCCAGAAGGCTTTGACT
1b      GAGTGCTGGGGTTCTGTCATTTCCAGCTCCCTGATGACCAGTTCGCTGAGGGCTTCCAGT
1c      GAGTGCTAGGGTTCTGTCATTTCCAGCTCCCTGATGACCAGTTCGCTGAGGGCTTCCAGT
        ******* ** ***** **************************  * ** *****  * *

1a      TTGACTGTGAAGATGTGAACTTTCCCACTGAGAATCTGTGCTTCGTTGGCCTCATGTCCA
1b      TTGATTGTGAAGAGGTGAACTTCCCAACTGAGAATCTGTGCTTCGTTGGCCTAATGTCCA
1c      TTGATTGTGAAGAGGTGAACTTCCCAACTGAGAATCTGTGCTTCGTTGGCCTAATGTCCA
        **** ******** ******** ** ************************** *******

1a      TGATTGACCCTCCCCGTGCTGCTGTGCCTGACGCTGTGAGCAAGTGCAGGTGTGCTGGAA
1b      TGATTGACCCTCCCCGTGCTGCTGTGCCTGATGCTGTGGGAAAGTGCAGGAGTGCTGGAA
1c      TGATTGACCCTCCCCGTGCTGCTGTGCCTGATGCTGTGGGAAAGTGCAGGAGTGCTGGAA
        ******************************* ****** * ********* *********

1a      TCAAGGTTATCATGGTCACTGGGGATCATCCCATCACTGCGAAGGCCATTGCCAAGGGTG
1b      TCAAGGTTATCATGGTCACTGGTGATCATCCCATCACTGCTAAGGCCATTGCCAAGGGTG
1c      TCAAGGTTATCATGGTCACTGGTGATCATCCCATCACTGCTAAGGCCATTGCTAAGGGTG
        ********************** ***************** *********** *******

1a      TGGGCATCATCTCTGAGGGCAACGAGACTGTTGAGGAAATTGCTGCTCGTCTGAAAATCC
1b      TGGGCATCATTTCTGAAGGAAACGAGACTGTTGAGGACATCGCTGCTCGTCTGAAAATCC
1c      TGGGCATCATTTCTGAAGGAAACGAGACTGTTGAGGACATTGCTGCTCGTCTGAACATTC
        ********** ***** ** ***************** ** ************** ** *
```

```
1a        CAGTTTCTGAGGTCAACCCAAGAGATGCCAAGGCCTGTGTTGTCCACGGTGGAGAGCTGA
1b        CAGTTTCTGAGGTCAATCCAAGAGATGCCAAGGCCTGTGTTGTCCACGGTGGAGAGCTGA
1c        CAGTCAATGAAGTTGACCCTAGGGATGCCAAGGCCTGTGTGGTTCATGGCGGTGACCTCA
          ****   *** **  * ** ** ***************** ** ** ** ** ** ** *

1a        AGGACATGACCCCCGAAGAGTTGGATGATATCCTGATGCATCACACTGAGATTGTGTTTG
1b        AGGACCTGTCAGCCGAACAGTTGGATGACATTTTGGCCCATCACACAGAGATTGTGTTTG
1c        AGGACCTGAGCGCTGAACAGTTGGACGACATCCTGAAATATCACACAGAGATTGTGTTTG
          ***** **    * *** ******* ** **  **    ******* *************

1a        CCAGAACTTCTCCTCAGCAGAAACTGATTATTGTGGAGGGTTGCCAGCGTCAGGGGGCCA
1b        CCAGAACATCTCCTCAGCAGAAGCTGATCATTGTGGAGGGTTGCCAGCGTCAGGGTGCTA
1c        CCAGAACCTCTCCTCAGCAGAAGCTTATTATCGTGGAGGGCTGCCAGCGCCAGGGTGCTA
          ******* ************** ** ** ** ******** ******** ***** ** *

1a        TTGTGGCTGTGACAGGCGATGGTGTGAACGATTCCCCTGCTCTGAGGAAAGCTGACATCG
1b        TTGTGGCTGTGACAGGTGATGGTGTGAACGATTCTCCTGCTCTGAAGAAGGCTGACATCG
1c        TTGTGGCTGTGACAGGTGATGGTGTGAACGATTCTCCTGCTCTGAAGAAGGCTGACATCG
          **************** ***************** ********** *** **********

1a        GTGTTGCTATGGGTATCGCTGGATCTGACGTCTCCAAGCAGGCTGCAGACATGATCCTCC
1b        GTGTTGCTATGGGGATCTCTGGATCTGACGTCTCCAAGCAGGCTGCAGACATGATCCTCC
1c        GTGTTGCTATGGGGATCTCTGGATCTGACGTCTCCAAGCAGGCTGCAGACATGATCCTCC
          ************* *** ******************************************

1a        TGGATGACAACTTTGCCTCCATCGTGACTGGTGTTGAAGAGGGCCGTCTGATCTTTGACA
1b        TGGATGACAACTTTGCCTCCATCGTGACTGGTGTTGAAGAGGGCCGTCTGATCTTTGACA
1c        TGGATGACAACTTTGCCTCCATCGTGACTGGTGTTGAAGAAGGCCGTCTGATCTTTGACA
          **************************************** *******************

1a        ACTTGAAGAAGTCCATCACCTACACCCTGTCCAGTAAAATTCCAGAGATGACCCCCTTCC
1b        ACTTGAAGAAGTCCATCGCCTACACCTTGACCAGTAATATTCCAGAAATCTCACCCTTCC
1c        ACTTGAAGAAGTCCATCGCCTACACCCTGACCAGTAACATTCCAGAAATCACACCCTTCC
          ***************** ******** ** ******* ******** **  * *******

1a        TCTTCTTGCTCCTCGCCAACATTCCACTGGCCCTAGGGACCGTCACCATACTCTGCATCG
1b        TCCTCTTCATCATCGCCAACATTCCACTGCCCCTAGGAACCGTCACCATCCTCTGTATCG
1c        TCTTCTTCATCATCGCCAACATTCCACTGCCCCTAGGAACCGTCACCATCCTCTGTATCG
          ** ****  ** ***************** ******* *********** ***** ****

1a        ACCTGGGAACTGACATGATCCCCGCTATCTCCCTGGCCTATGAACAAGCTGAGAACGACA
1b        ACCTGGGAACTGATATGGTCCCAGCTATCTCCCTGGCCTATGAAGAAGCTGAGAATGACA
1c        ACCTGGGAACTGACATGGTCCCCGCCATCTCCCTGGCCTACGAAGCTGCTGAGAGTGACA
          ************* *** **** ** ************** ***   *******  ****

1a        TCATGAAGAGACAGCCACGGAACCCCAAAACAGACCGACTGGTG AACGAGAGACTCATTA
1b        TTATGAAGCGACAGCCCAGAAACCCCAAAACTGACAAACTGGTG AACGAGAGGCTCATTA
1c        TCATGAAGAGACAGCCCAGAAACTCCAAAACAGACAAACTGGTG AACGAAAGACTTATCA
          * ****** *******  * *** ******* ***  ******* ***** ** ** ** *

1a        GTGTGGCCTATGGTCAATTTGGAGTGATGCTGGCCGCAGCC GGCTTCTTCACATACTTTG
1b        GCATAGCCTACGGTCAAATTGGTATGATGCAGGCCACAGCC GGTTTCTTCACATACTTTG
1c        GCATAGCCTACGGTCAAATCGGTATGATCCAGGCTTTGGCT GGGTCCTTCACATACTTTG
          *  * ***** ****** * **  **** * ***    **  ** * **************

1a        TAATCATGGCTGAGAACGGGTTCTATCCCATGGACTTGCTAGGAATCCGTTTGGACTGGG
1b        TTATCCTGGCTGAGAACGGGTTCTTGCCCATGGATTTGCTGGGTATGCGTGTGGACTGGG
1c        TGATCCTTGCTGAGAACGGGTTCTTACCCTCCAGACTGCTAGGTATTCGTGTGGACTGGG
          * *** * ****************  ***       **** ** ** *** *********

1a        AAAACCAGTACATCAACGACTTGGAGGACAGCTACGGCCAGCAGTGGACATATGAGAGCA
1b        ACAACAAGATAATGAACGACATGGAGGACAGCTACGGCCAGCAGTGGACATATGAGCGCA
1c        ACAACAAATTTTGCAACGACCTGGAGGATAGCTATGGCCAGCAGTGGACTTATGAACAGA
          * *** *       ****** ******* ***** ************** *****    *
```

```
1a      GAAAGATAATTGAGTTCACCTGCCACACAGCGTACTTCGCCGCTGTTGTGATTGCACAGT
1b      GAAAGATTGTGGAGTTCACCTGCCACACAGCATTCTTCGCCAGTATTGTAGTTGTACAGT
1c      GAAAGATTGTGGAGTTCACCTGCCACACAGCATTCTTCGCCAGTATTGTAGTTGTACAGT
        *******  * ******************** * *******  * ****  *** *****

1a      GGGCCGTTTTGATCGTCTGTAAGACCAGGAAGAACTCCTTC TTTCAGCAGGGACTAATGA
1b      GGGCTGATTTGATCATCTGTAAGACCAGGAGGAACTCCATC CTTCAGCAGGGAAT---GA
1c      GGGCTGATTTGATCATCTGTAAGACCAGGAGGAACTCAGTC TTCCAACAAGGAAT---GA
        **** * ******* *************** ******  **  * ** ** *** *   **

1a      AGAACCGTGTTCTCATCTTCGGACTTTGTTCAGAAT CCGCCCTGGCTCTCTTCCTGTCCT
1b      AGAACCGTATTCTCATCTTCGGACTGTTTGAGGAAA CTGCCCTGGCTGTCTTCCTGTCCT
1c      GGAACAAGATTCTCATCTTCGGCCTGCTTGAGGAGA CCGCCCTGGCCGCCTTCCTGTCCT
         ****    ************* **   *   **   * ********   ***********

1a      ACTGCCCTGGAATGGACGTTGCCATCAGAATGTACCCACTCAAGCCTTTCTGGTGGGTAT
1b      ACTGTCCTGGAATGGATGTTGCCCTCAGAATGTACCCCCTCAAGCCTTGCTGGTGGTTCT
1c      ACTGTCCTGGGATGGGCATCGTCCTCAGAATGTACCCACTCAAACCCAGCTGGTGGTTCT
        **** ***** ****   * * * ************* ***** **   ******* * *

1a      GTGCCTTTCCCTACACCCTGCTCATCTT CATCTATGATGAGGTTAGAAAATACATCATGC
1b      GTGCCTTACCCTACTCTCTGCTCATCTT CCTCTATGATGAGGGTAGGAGATACATCCTGC
1c      GCGCCTTCCCATACTCTCTCCTCATCTT TATTTATGATGAAATCCGAAAACTGATCATCC
        * ***** ** *** * ** ********   * ********     * * *   *** * *

1a      GACGGAACTCAGGAGGTTGG GTGTACCAAGAGACATACTATTGAAACGAAGAGGTCGGGA
1b      GACGAAACCCAGGAGGTTGG GTGGAACAGGAGACCTACTACTGACAAAAAGAGGCTATCC
1c      GACGCAGCCCAGGAGGTTGG GTGGAGAGGGAGACGTACTACTAGAAAGCATCCCGTTTGC
        **** * * *********** *** *    ***** ***** *   *   *

1a      ATGGTCTCTTCT TTCAGGTTGGGTGTACCAAGAGACATACTATTGAAACGAAGAGGCCGG
1b      CCAGGATCACTC AACATCACTCTGCTGCTACATCTCAACCAACAACATTACACTACATTT
1c      ATCGCTCAAGTT CTTGCA-TGGTTGTCTTGCTGCTCGGAATTTTAAATTCTGTAAAATGT
           *                      *         *          *

1a      GAATTGTCTCTTCTTTCAGGTTGGGTGTACCAAGAGACATACTAT---------TGAAAC
1b      CAATCTGCTACA--------------TTACCATTACACCCAATGAACACTGTGATGGTGC
1c      GGATGTGTTTTTATATAGGGAATGCTTGATACTAAAACACTGAGATCGAGATGATGAACC
          **    *                   *     * **                **   *

1a      AAAGAGGTCGGG-AATGGTCTCTTCTTTCAGGTTGGGTGTACCAAGAGACATACTATTGA
1b      ACTCAGCTGCATTGAGGAGTTCTTGATTGTGAT----AATGCAAATTGAAATAAACATCA
1c      AAGACATTTGACATTGTGTGCCTTGTTTCTGGA----AC-AGGACCAATTTTATACATGT
        *      *             ***  **  *            *       **    *

1a      AACGAAGAGGCCGTCCCAGGATCACTCAGTCACTCTGCTGCAGTATTGCCTTGTAATGCA
1b      CTTCAACAC----T----------------------------------------------
1c      TTTTAACAG----TAATATAAATAAACTTTCAGTCAGCTCCCG-----------------
            ** *     *

1a      ATCGTTCATTTTTACAATGATATTAAACCCATTTGCCTTTGATT
1b      --------------------------------------------
1c      --------------------------------------------
```
