# Supplementary figures and images for: Subcellular localization of Na+/K+-ATPase isoforms resolved by in situ hybridization chain reaction in the gill of chum salmon at freshwater and seawater
Source: Fish Physiol Biochem. 2023 Jul 19;49(4):751–67. doi: 10.1007/s10695-023-01212-6 (PMC10415477; doi:10.1007/s10695-023-01212-6)

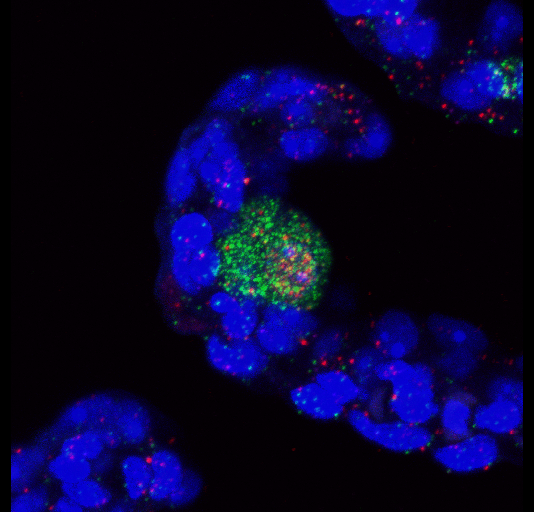

Supplement: Supplementary file 2 — Supplementary File 1. Z-stacked composite image of a Type-I ionocyte in the gill of freshwater (FW) chum salmon. When the file is opened with ImageJ program, the 3-dimensional image of the NKA α1a (green), α1b (red), and α1c (magenta) of the Type-I ionocyte can be observed. Nucleus is stained with Hoechst (blue). (TIF 28842 kb) [file 10695_2023_1212_MOESM2_ESM.tif]

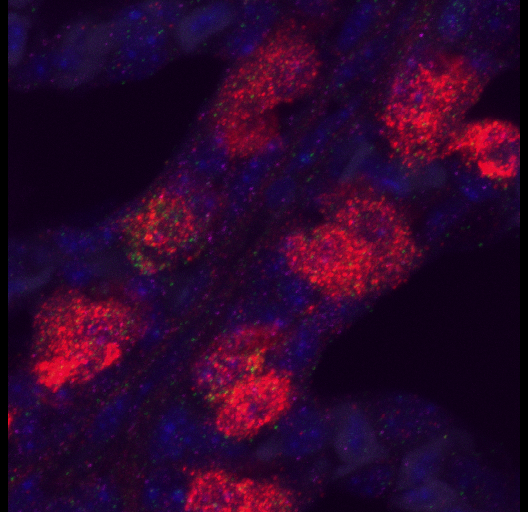

Supplement: Supplementary file 3 — Supplementary File 2. Z-stacked composite image of a Type-II ionocyte in the gill of seawater (SW) chum salmon. When the file is opened with ImageJ program, the 3-dimensional image of the NKA α1a (green), α1b (red), and α1c (magenta) of the Type-II ionocyte can be observed. Nucleus is stained with Hoechst (blue). (TIF 28518 kb) [file 10695_2023_1212_MOESM3_ESM.tif]
