# Supplementary material for: Subcellular localization of Na+/K+-ATPase isoforms resolved by in situ hybridization chain reaction in the gill of chum salmon at freshwater and seawater
Source: Fish Physiol Biochem. 2023 Jul 19;49(4):751–67. doi: 10.1007/s10695-023-01212-6 (PMC10415477; doi:10.1007/s10695-023-01212-6)
Supplement: Supplementary file 4 — Supplementary Table 1. Split probes for NKA α1-isoforms in chum salmon. Initiator sequences for hairpin are underlined. (DOCX 17 kb) [file 10695_2023_1212_MOESM4_ESM.docx]

Supplementary Table 1. Split probes for NKA α1-isoforms in chum salmon. Initiator sequences for hairpin are underlined.

|  | Primer name | Nucleotide Sequences |
| --- | --- | --- |
| **α1a** |  |  |
| Set 1 | NKA1a_F1 | CCTCCACGTaaTTTCTGAAATGATTCCTTTATCTCG |
| (#45) | NKA1a_R1 | TCCCAGTCCTCCTAGCGCTTCGTAGaaTCCATCTAAGCT |
|  |  |  |
| Set 2 | NKA1a_F2 | CCTCCACGTaaAGGCCACACTAATGAGTCTCTCGTT |
|  | NKA1a_R2 | CGGCCAGCATCACTCCAAATTGACCaaTCCATCTAAGCT |
|  |  |  |
| Set 3 | NKA1a_F3 | CCTCCACGTaaGGTTCTTCATTAGTCCCTGCTGAAA |
|  | NKA1a_R3 | CTGAACAAAGTCCGAAGATGAGAACaaTCCATCTAAGCT |
|  |  |  |
| Set 4 | NKA1a_F4 | CCTCCACGTaaGTATTTTCTAACCTCATCATAGATG |
|  | NKA1a_R4 | CCAACCTCCTGAGTTCCGTCGCATGaaTCCATCTAAGCT |
|  |  |  |
| Set 5 | NKA1a_F5 | CCTCCACGTaaTTCAATAGTATGTCTCTTGGTACAC |
|  | NKA1a_R5 | AGAAGAGACCATTCCCGACCTCTTCaaTCCATCTAAGCT |
|  |  |  |
| **α1b** |  |  |
| Set 1 | NKA1b_F1 | GCTCGACGTaaGTTCTGGAAGGCTTCCTTCAGCTCG |
| (#41) | NKA1b_R1 | TCCCAGCCCTCCCAGCTCTTCATAAaaTCCTTTGCAACA |
|  |  |  |
| Set 2 | NKA1b_F2 | GCTCGACGTaaACCGTAGGCTATGCTAATGAGCCTC |
|  | NKA1b_R2 | GGCTGTGGCCTGCATCATACCAATTaaTCCTTTGCAACA |
|  |  |  |
| Set 3 | NKA1b_F3 | GCTCGACGTaaTACGGTTCTTCATTCCCTGCTGAAG |
|  | NKA1b_R3 | TTTCCTCAAACAGTCCGAAGATGAGaaTCCTTTGCAACA |
|  |  |  |
| Set 4 | NKA1b_F4 | GCTCGACGTaaGTATCTCCTACCCTCATCATAGAGG |
|  | NKA1b_R4 | CCAACCTCCTGGGTTTCGTCGCAGGaaTCCTTTGCAACA |
|  |  |  |
| Set 5 | NKA1b_F5 | GCTCGACGTaaGTCAGTAGTAGGTCTCCTGTTCCAC |
|  | NKA1b_R5 | GAGTGATCCTGGGGATAGCCTCTTTaaTCCTTTGCAACA |
|  |  |  |
| **α1c** |  |  |
| Set 1 | NKA1c_F1 | CGGTCAGGTaaGTTCTGGAAAGAGTCCTTCATCTCG |
| (#73) | NKA1c_R1 | TCCCAGACCACCCAGTTCCATGTAGaaGGCTAGTATGGA |
|  |  |  |
| Set 2 | NKA1c_F2 | CGGTCAGGTaaCCGTAGGCTATGCTGATAAGTCTTT |
|  | NKA1c_R2 | GCCAAAGCCTGGATCATACCGATTTaaGGCTAGTATGGA |
|  |  |  |
| Set 3 | NKA1c_F3 | CGGTCAGGTaaTCTTGTTCCTCATTCCTTGTTGGAA |
|  | NKA1c_R3 | TCTCCTCAAGCAGGCCGAAGATGAGaaGGCTAGTATGGA |
|  |  |  |
| Set 4 | NKA1c_F4 | CGGTCAGGTaaCAGTTTTCGGATTTCATCATAAATA |
|  | NKA1c_R4 | CCAACCTCCTGGGCTGCGTCGGATGaaGGCTAGTATGGA |
|  |  |  |
| Set 5 | NKA1c_F5 | CGGTCAGGTaaTCTAGTAGTACGTCTCCCTCTCCAC |
|  | NKA1c_R5 | AACTTGAGCGATGCAAACGGGATGCaaGGCTAGTATGGA |
|  |  |  |
